# 305. Cholecystitis as a Possible Immunologic Consequence of COVID-19; Case Series from a Large Healthcare System

**DOI:** 10.1093/ofid/ofab466.507

**Published:** 2021-12-04

**Authors:** Anna Jacobs, Christopher Polk, Mindy Sampson, Banks Kooken, Thomas Ludden, Catherine Passaretti, Catherine Passaretti, Michael Leonard

**Affiliations:** 1 Carolinas Medical Center - Atrium Health, Charlotte, North Carolina; 2 Atrium Health, Charlotte, North Carolina; 3 UNC School of Medicine, Charlotte, North Carolina

## Abstract

**Background:**

Gastrointestinal manifestations are commonly seen in COVID-19 disease with up to 50% of patients reporting nausea or diarrhea. Cholecystitis has been described in rare cases related to COVID-19, possibly in consequence of immune activation, but biliary disease from SARS-CoV-2 infection is not well described. We examined a case series of patients with both COVID-19 and cholecystitis at our institution.

**Methods:**

We performed a retrospective chart review of all patients with a diagnosis of cholecystitis within 3 months of SARS-CoV-2 infection; looking at clinical, laboratory, and radiographic characteristics of this population.

**Results:**

30 individuals were identified with a diagnosis of cholecystitis within 3 months of diagnosis of SARS-CoV-2 infection. Most patients presenting with cholecystitis were female and obese (see Table 1). 14 individuals were diagnosed with SARS-CoV-2 infection during the same presentation as their cholecystitis diagnosis, usually as part of pre-operative screening. Of 16 individuals diagnosed with SARS-CoV-2 prior to their cholecystitis presentation, a mean of 24 and 17 days elapsed between SARS-CoV-2 infection and cholecystitis symptom onset and radiographic diagnosis, respectively (see Figure 1). Most of these patients had mild respiratory disease, with only 9 developing an oxygen requirement, and only 3 requiring mechanical ventilation. While 17 patients were treated surgically for their cholecystitis, this did not appear to impact symptom resolution.

Table 1. Patient Characteristics

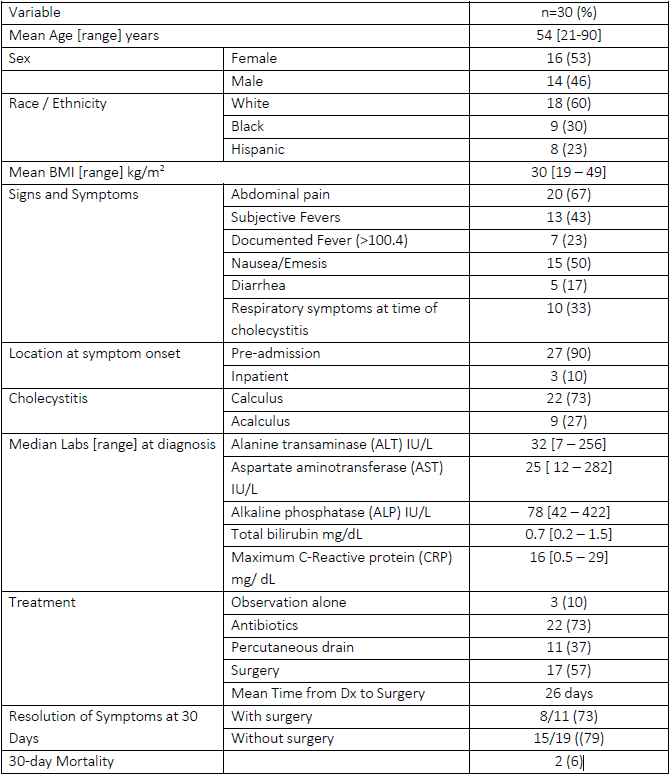

Figure 1. Time between COVID-19 and Cholecystitis

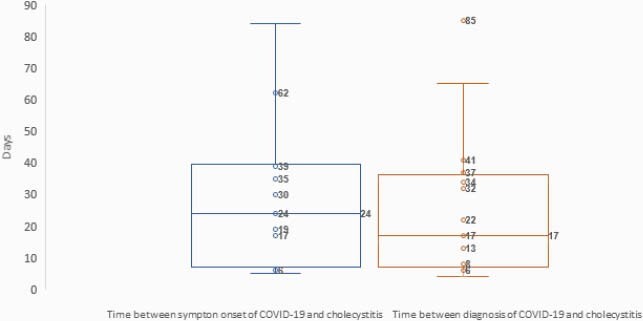

**Conclusion:**

Cholecystitis may be an uncommon complication of COVID-19 disease. Cholecystitis may manifest most often 2-4 weeks following SARS-CoV-2 infection. This timing is similar to that in Multisystem Inflammatory Syndrome following SARS-CoV-2 infection and given similarities in timing to we hypothesize that cholecystitis in our patients could be driven by immune activation.

**Disclosures:**

**Christopher Polk, MD**, **Atea** (Research Grant or Support)**Gilead** (Advisor or Review Panel member, Research Grant or Support)**Humanigen** (Research Grant or Support)**Regeneron** (Research Grant or Support) **Mindy Sampson, MD**, **Regeneron** (Grant/Research Support) **Catherine Passaretti, MD**, Nothing to disclose

